# Silencing of Hypoxia-Inducible Factor-1β Induces Anti-Tumor Effects in Hepatoma Cell Lines under Tumor Hypoxia

**DOI:** 10.1371/journal.pone.0103304

**Published:** 2014-07-28

**Authors:** Sung Hoon Choi, Ae Ri Chung, Wonseok Kang, Jun Yong Park, Mi Sol Lee, Shin Won Hwang, Do Young Kim, Seung Up Kim, Sang Hoon Ahn, Seungtaek Kim, Kwang-Hyub Han

**Affiliations:** 1 Brain Korea 21 plus project for Medical Science, Yonsei University College of Medicine, Seoul, Korea; 2 Department of Premed, Yonsei University College of Medicine, Seoul, Korea; 3 Department of Internal Medicine, Yonsei University College of Medicine, Seoul, Korea; 4 Yonsei Liver Cancer Special Clinic, Yonsei University Health System, Seoul, Korea; 5 Liver Cirrhosis Clinical Research Center, Yonsei University Health System, Seoul, Korea; Konkuk University, Republic Of Korea

## Abstract

Dimerization of hypoxia-inducible factor-1 beta (HIF-1β) [aryl hydrocarbon receptor nuclear translocator (ARNT)] with HIF-1α is involved in various aspects of cancer biology, including proliferation and survival under hypoxic conditions. We investigated the *in vitro* mechanism by which silencing of HIF-1β leads to the suppression of tumor cell growth and cellular functions. Various hepatocellular carcinoma (HCC) cell lines (Huh-7, Hep3B, and HepG2) were transfected with small interfering RNA (siRNA) against HIF-1β (siHIF-1β) and cultured under hypoxic conditions (1% O_2_ for 24 h). The expression levels of HIF-1β, HIF-1α, and growth factors were examined by immunoblotting. Tumor growth was measured using the 3-(4,5-dimethylthiazol-2-yl)-2,5-diphenyltetrazolium bromide assay, and tumor activity was measured by terminal deoxynucleotidyl transferase dUTP nick end labeling, tumor cell invasion, and migration assays. Under hypoxic conditions, silencing of HIF-1β expression suppressed tumor cell growth and regulated the expression of tumor growth-related factors, such as vascular endothelial growth factor, epidermal growth factor, and hepatocyte growth factor. Suppression of tumor cell invasion and migration was also demonstrated in HIF-1β-silenced HCC cell lines. Silencing of HIF-1β expression may induce anti-tumor effects under hypoxic conditions in HCC cell lines.

## Introduction

Tumor hypoxia is one of the distinguished features of tumor microenvironment found in hepatocellular carcinoma (HCC) [1], [2]. Hypoxic microenvironment usually occurs in a rapidly proliferating tumor, mainly due to its increased metabolic rate and oxygen consumption [3], [4]. Hypoxia plays an important role in tumor progression through angiogenesis and resistance to apoptosis [2]. Cellular response to hypoxia is mediated, at least in part, by a family of transcription factors known as hypoxia-inducible factors (HIFs) [2], [3].

HIF-1 is a heterodimeric transcription factor, and is composed of two subunits, the oxygen-sensitive HIF-1α and constitutively expressed HIF-1β (also called the aryl hydrocarbon receptor nuclear translocator (ARNT) [5], [6]. Under low oxygen tension, the activated transcription factor, HIF-1α, upregulates diverse hypoxia-inducible genes through dimerization with HIF-1β, a co-activator of HIF-1β, and binds to the hypoxia-responsive element in the promoter of target genes [5], [6].

HIF-1β plays an important role in the differentiation and development of many cells including T-cells, neurons, and hepatocytes under normoxia [5]. HIF-1β together with HIF-α, forms a complex with many other proteins and participates in various functions in the cell [7], [8]. It is also a co-activator of several activators, and plays the role of receptor and transcription factor by connecting activators [6], [9]. Similar to HIF-1α-up regulation, the expression of HIF-1β is increased by approximately two folds under hypoxic conditions [10]. The interaction of HIF-1α and HIF-1β is critical in the process of tumor survival. Although the influences of HIF-1α on tumor cells have been widely studied [3], the role of HIF-1β expression in tumor cell survival have been reported in few literature and therefore remains to be investigated. In this study, we demonstrated the effects of silencing HIF-1β expression on HCC cells, and found that HIF-1β-silencing regulates dimerization with HIF-1α under hypoxic conditions, leading to the suppression of tumor cell growth, invasion, and migration.

## Materials and Methods

### Cell culture

Huh-7(KCLB60104, Korean Cell line Bank), Hep3B [11], and HepG2(KCLB88065, Korean Cell line Bank) cells were cultured at 37°C with 5% CO_2_ in Dulbecco’s Modified Eagle Medium (DMEM; Gibco, Grand Island, NY) supplemented with 10% fetal bovine serum (FBS; Welgene, Daegu, Korea), 4.5 g/L glucose, L-glutamine, and 1% penicillin/streptomycin.

### small interfering RNA (siRNA) and transfection

siRNA was synthesized using the following sequences: siHIF-1α: (Forward) 5′-GUG GUU GGA UCU AAC ACU A-3′, (Reverse) 5′-UAG UGU UAG AUC CAA CCA C-3′; siHIF-1β: (Forward) 5′-CAG ACA AGC UAA CCA UCU U-3′, (Reverse) 5′-AAG AUG AGC UUG UGU U-3′. Cells were transfected with respective siRNAs using Fugene HD transfection agent (Promega, Madison, WI, USA) according to the manufacturer’s instructions. After transfection, the cells were maintained at normoxic conditions (21% O_2_) for 24 h, then replaced with fresh culture medium, followed by 24 h of culture under hypoxic conditions (1% O_2_, 5% CO_2_, 94% N_2_)^8^.

### Cell growth assay

Tumor cell growth rate was measured by the 3-(4,5-dimethylthiazol-2-yl)-2,5-diphenyltetrazolium bromide (MTT; Amresco, Solon, OH, USA) assay according to the manufacturer’s instructions. Briefly, the cells were seeded in a 96-well plate at 5×10^3^ cells/well, incubated at 37°C for 24 h, transferred to serum-free medium, and transfected with siRNA. After incubation at 21% O_2_ for 24 h, the culture medium was replace with fresh medium supplemented with 10% FBS. Hypoxia was induced by incubation under 1% O_2_ at 37°C for 24 h. After addition of MTT to each well, the plates were incubated at 37°C for 3∼4 h for sufficient staining of cells, followed by medium removal and dimethylsulfoxide (Sigma-Aldrich, St. Louis, MO, USA) treatment. Cell viability was measured with absorbance at 595 nm using a spectrophotometer (Molecular Devices, Toronto, Canada).

### Real-time quantitative polymerase chain reaction (PCR)

Total RNA was extracted from HCC cells using Trizol reagent (GIBCO BRL, Grand Island, NY, USA) according to the manufacturer’s instructions. cDNA was synthesized from 1 µg of RNA using reverse transcriptase (Clontech, Mountain View, CA, USA), according to the manufacturer’s instructions. Gene expression was measured by real-time quantitative PCR.

### Immunoblot assay

The effects of silencing HIF-1α and HIF-1β expression on the expression of proteins related to cell proliferation and angiogenesis were assessed by immunoblot assays. Total proteins were extracted from HIF-1α- or HIF-1β-silenced HCC cells after 24 h of hypoxia induction. The proteins were separated according to their molecular weight via sodium dodecyl sulfate–polyacrylamide gel electrophoresis (SDS-PAGE), transferred to a polyvinylidene fluoride membrane (GE Healthcare/Amersham, Buckinghamshire, UK), and blotted with mouse monoclonal antibodies specific for the proteins of interest. The blots were developed using the enhanced chemiluminescence technique (PerkinElmer, Boston, MA, USA) according to the manufacturer’s instructions, and the level of expression of each protein was quantified and compared. For detection of secreted proteins, enzyme-linked immunosorbent assay (ELISA; R&D Systems, Minneapolis, MN, USA) was performed according to the manufacturer’s instructions.

### Immunoprecipitation

Total cell lysate was extracted from HCC cells using RIPA cell lysis buffer (Cell signaling, Danver, MA, USA) according to the manufacturer’s instructions. HIF-1α was bounded with mouse anti-HIF-1α (Cell signaling, Danver, MA, USA) using IgG magnetic beads (Novex, Oslo, Norway), according to the manufacturer’s instructions. The dimerized proteins were detected according to western blot, and blotted with the other target-mouse monoclonal antibodies-HIF-1α (Bethyl, Montgomery, TX, USA) specific for the proteins of interest. The blots were developed using the enhanced chemiluminescence technique (PerkinElmer, Boston, MA, USA) according to the manufacturer’s instructions, and the level of expression of each protein was quantified and compared.

### Tumor cell invasion assay

The effects of HIF-1α and HIF-1β knockdown on tumor cell invasiveness were investigated in Huh-7 cells grown in serum-free DMEM. The invasiveness of tumor cells was assessed *in* *vitro* using a transwell chamber (Corning Costar, Cambridge, MA, USA). Each transwell chamber was plated with 1×10^5^ cells, and the invading cells were stained with hematoxylin and eosin. The total number of invaded cells on the lower side of the filter was counted under the microscope (Olympus America, Melville, NY, USA).

### Migration assay

The mobility of cells was assessed by scratch and wound healing assay. Each experimental result was observed by optical microscope.

### Cell death assay

Cells were stained with FITC-labeled annexin V and propidium iodide (PI), and tumor cell death was assessed by terminal deoxynucleotidyl transferase dUTP nick end labeling (TUNEL, Promega) assay and flow cytometry.

### Statistical analysis

Results were expressed as means ± standard error of the mean (SEM) or frequency (%). Independent t-test was performed to compare the difference of the mean between control and experimental groups. All statistical analysis was done using SPSS version 12.0 (SPSS, Inc., Chicago, IL). A *p* value of less than 0.05 was considered statistically significant.

## Results

### Silencing of HIF-1α and HIF-1β suppresses tumor cell growth

**Figure 1:**
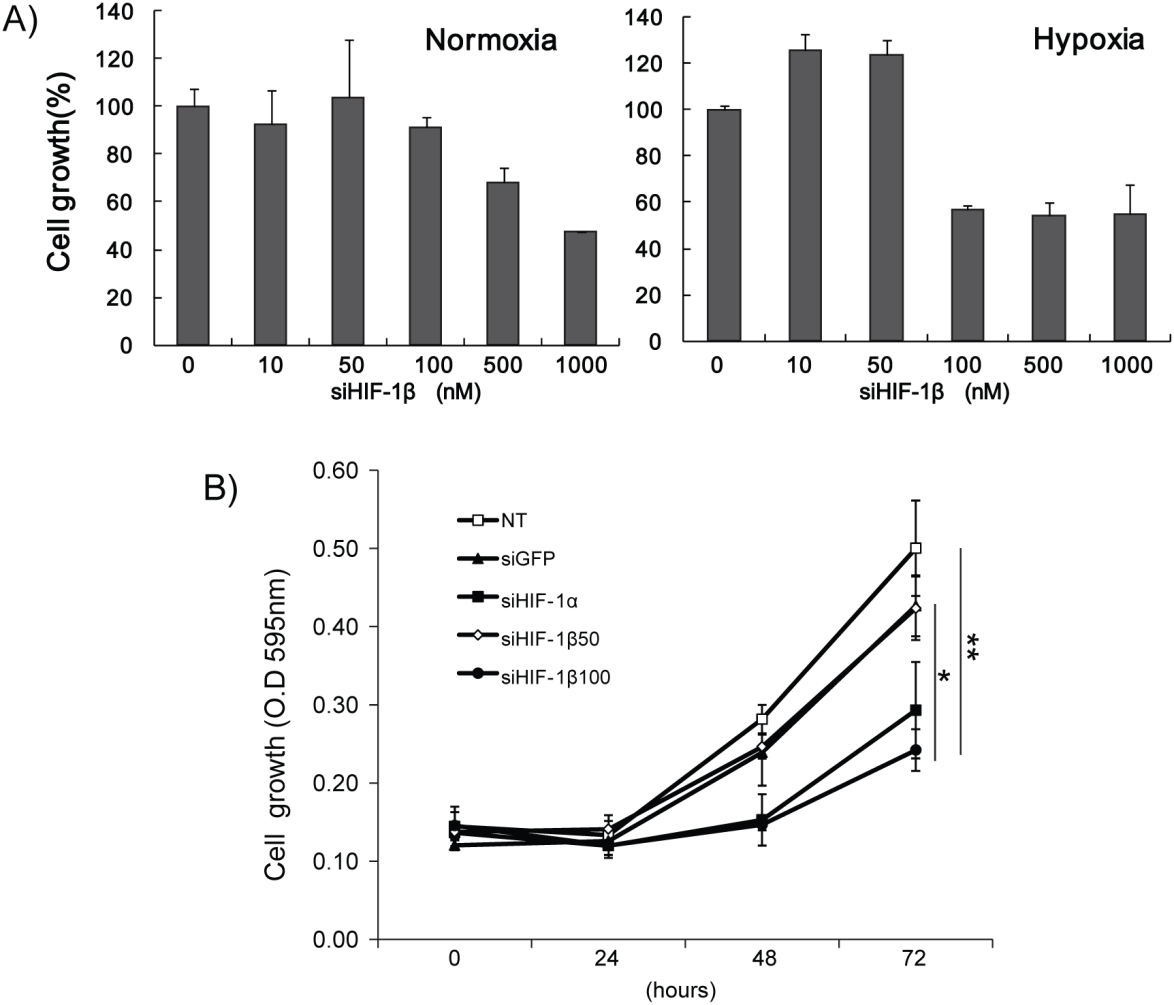
Suppression of tumor cell growth by silencing of hypoxia-inducible factors-1α and -1β. Huh-7 cells were transfected with small interfering RNAs against HIF-1α, HIF-1β, or green fluorescent protein as control (siHIF-1α, siHIF-1β, and siGFP, respectively), followed by exposure at hypoxic conditions (1% O_2_). (A) Tumor cell growth after silencing of HIF-1α or **-**1β was measured by 3-(4,5-dimethylthiazol-2-yl)-2,5-diphenyltetrazolium bromide (MTT) assay. Tumor cells were susceptible to growth retardation under hypoxic conditions when more than 100 nM of siHIF-1β was transfected. However, normoxic conditions (100 nM) did not show significant difference. (B) Transfection of siHIF-1α (100 nM) or siHIF-1β (100 nM) suppressed cell growth when maintained at hypoxic conditions. As compared to control, the growth inhibition was more prominent with increase of siHIF-1β concentration. NT, non-target; siGFP, siRNA against green fluorescent protein; siHIF1-α, siRNA against HIF-1α; siHIF-1β, siRNA against HIF-1β. *, *P*<0.05; **, *P*<0.01.

After transfection of HCC cells with various concentrations of siHIF-1β, tumor cell growth was assessed by MTT assay. Forty-eight hours after transfection, tumor cell growth was significantly suppressed as compared to control, in a dose-dependent manner. The negative effect of HIF-1β silencing on tumor cell growth was more prominent under hypoxic conditions, especially when more than 100 nM of siHIF-1β or siHIF-1α was transfected to the tumor cells (Fig. 1A, and File S1). Although the tumor cell growth was maintained at higher doses of siHIF-1β transfection under normoxic conditions, exposure to hypoxic environment resulted in significant suppression of tumor cell growth. Suppression of tumor cell growth under hypoxic conditions by HIF-1β-silencing was more pronounced by prolonged exposure to hypoxic environment. (Fig. 1B). Of note, consistent with the previous findings [12], silencing of HIF-1α also displayed suppression of tumor cell growth under hypoxic conditions, which is probably mediated by inhibition of several other targets related to cell proliferation. These findings demonstrate that tumor cell growth is suppressed by silencing of HIF-1β under hypoxic conditions.

### Silencing of HIF-1β affects expression of tumor growth-related genes

**Figure 2:**
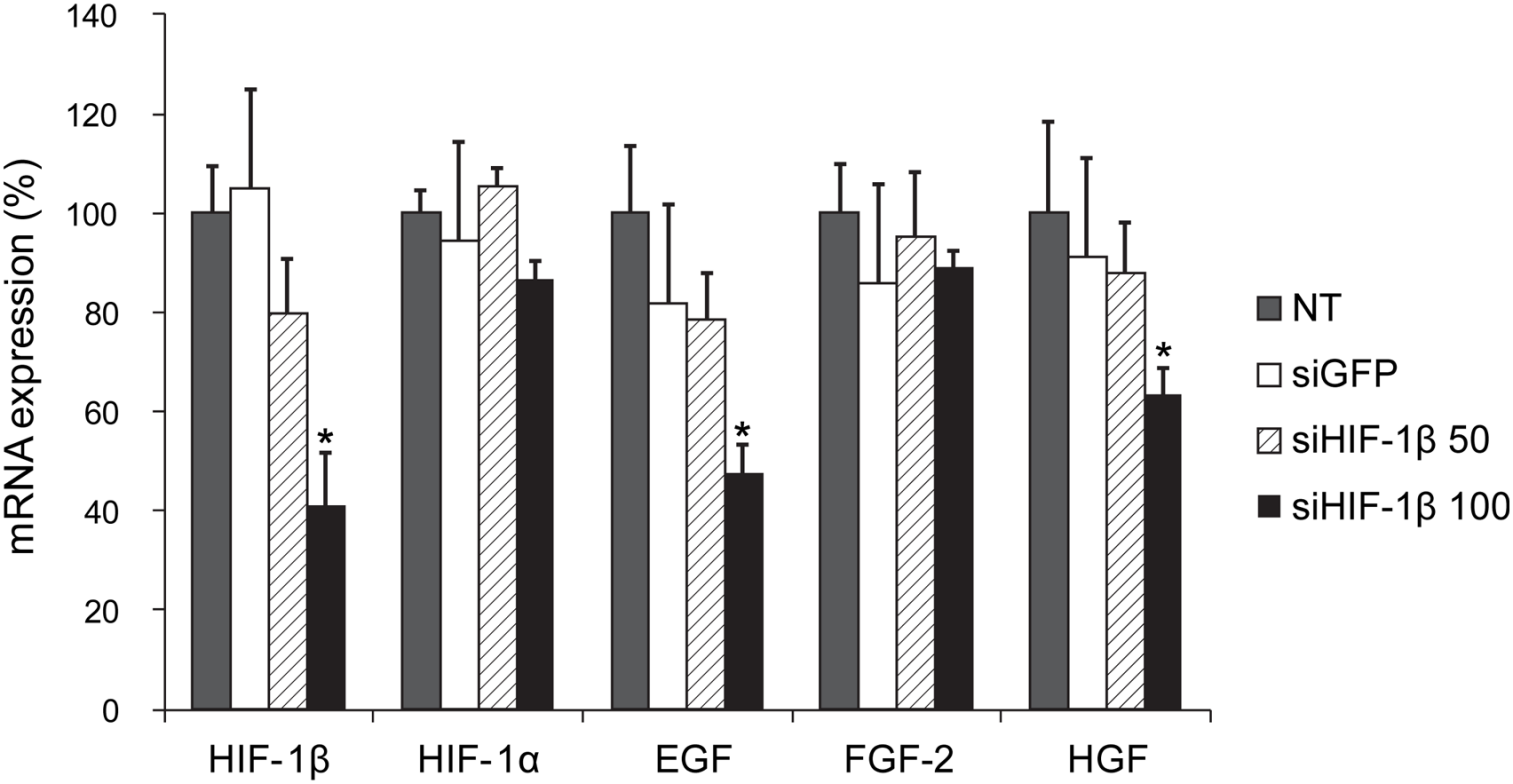
Silencing of HIF-1β affects expression of tumor growth-related genes. Under hypoxic conditions, silencing of HIF-1β was associated with diminished expression of several genes related to tumor growth, such as EGF and HGF, but not FGF2. NT, non-target; siGFP, siRNA against green fluorescent protein; siHIF1-α, siRNA against HIF-1α; siHIF-1β, siRNA against HIF-1β; EGF, epidermal growth factor; HGF, hepatocyte growth factor; FGF2, fibroblast growth factor 2. *, *P*<0.05.

Since tumor cell growth was inhibited by HIF-1β-silencing, we measured the mRNA levels of growth factors involved in tumor cell growth in HIF-1β-silenced tumor cells. Expression of HIF-1β was reduced by >60% in the group treated with siHIF-1β compared with that in the Control group, in a cell-density-dependent manner. Therefore, siHIF-1β inhibits expression of HIF-1β. HIF-1α expression was induced by hypoxia regardless of HIF-1β knockdown. To determine the influence of HIF-1β knockdown on genes related to tumor growth, the mRNA expression levels of epidermal growth factor (EGF), fibroblast growth factor (FGF), and hepatocyte growth factor (HGF) were quantified by real-time quantitative PCR (Fig. 2). Under hypoxic conditions, silencing of HIF-1β produced diminished expression of EGF and HGF by 52% and 36% (p-value<0.05), respectively, compared to control. However, mRNA levels of FGF2 expression was not affected by HIF-1β-silencing. Collectively, these data suggest that under hypoxic conditions, HIF-1β expression regulates the expression of tumor growth-related factors, namely EGF and HGF, but not FGF2.

### Silencing of HIF-1β affects protein expression and secretion of tumor growth-related genes

**Figure 3:**
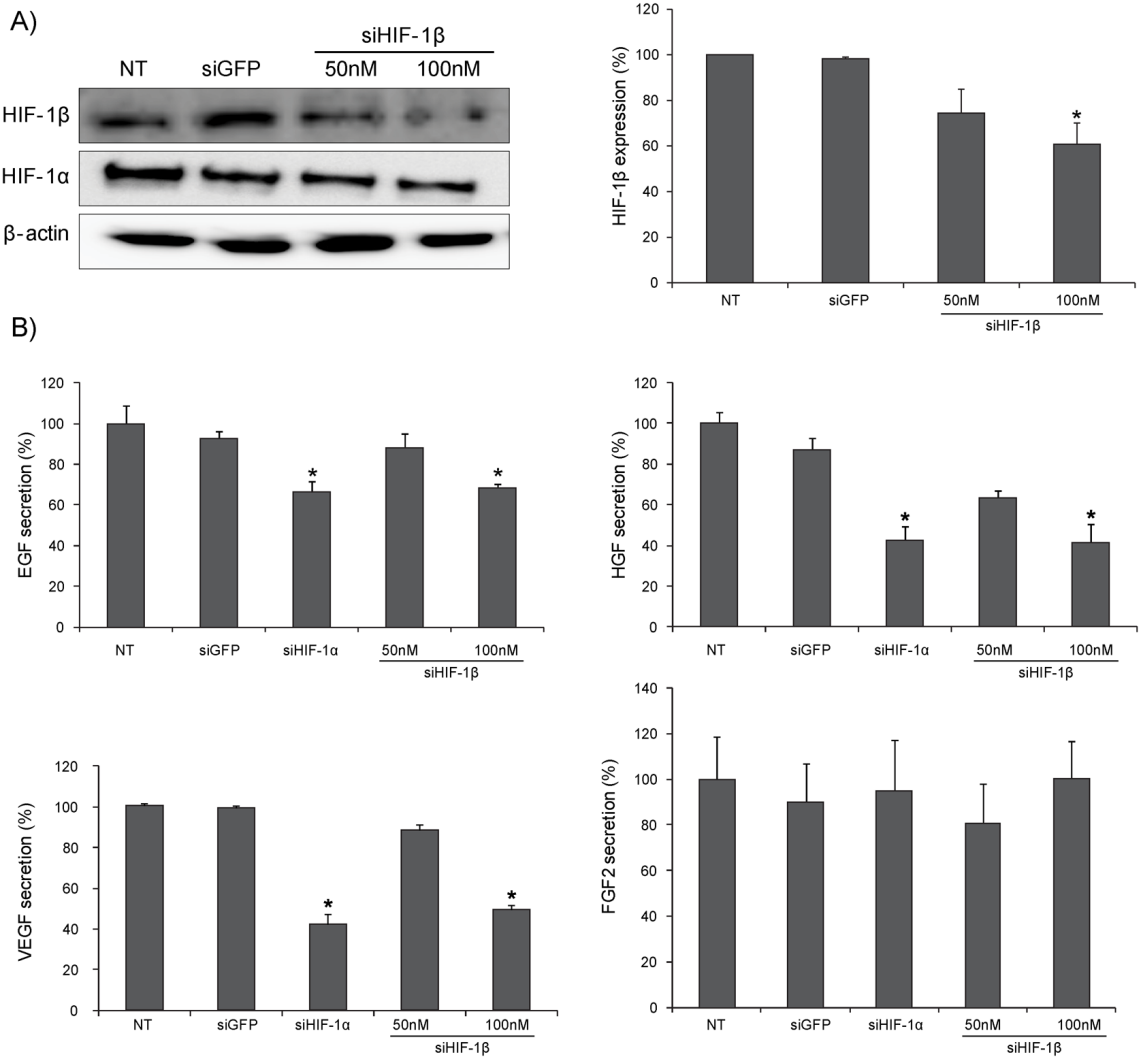
Silencing of HIF-1β affects protein expression and secretion of tumor growth-related genes. (A) Selective silencing of HIF-1β protein expression by siHIF-1β was confirmed by immunoblot assays. (B) Decreased expression and secretion of EGF, HGF, and VEGF by silencing of HIF-1α and **-**1β was confirmed by enzyme-linked immunosorbent assay. NT, non-target; siGFP, siRNA against green fluorescent protein; siHIF1-α, siRNA against HIF-1α; siHIF-1β, siRNA against HIF-1β; EGF, epidermal growth factor; HGF, hepatocyte growth factor; VEGF, vascular endothelial growth factor. *, *P*<0.05.

Based on the aforementioned mRNA data, protein expression of tumor growth-related genes were analyzed using Hep3B cells. Dose-dependent and selective inhibition of HIF-1β expression by siHIF-1β transfection was confirmed by immunoblot assays (Fig. 3A). Interaction of HIF-1β and HIF-1α was confirmed by immunoprecipitation (File S2). IP results demonstrated that decreased protein expression levels of HIF-1β or HIF-1α in HIF-1α tagged group. To determine the influence of HIF-1β inhibition on factors related to tumor cell growth, the levels of protein expression and secretion of EGF, HGF, VEGF, and FGF2 were analyzed (Fig. 3B). ELISA results demonstrated that decreased protein expression levels of EGF, HGF, and VEGF in HIF-1β-silenced cells. Of note, the expression of these molecules was also decreased in HIF-1α-silenced cells. Notably, the expression of FGF2 was not affected by silencing of HIF-1α or HIF-1β. Collectively, these results demonstrate that under hypoxic conditions, HIF-1β expression regulates the expression of various tumor growth-related factors, namely EGF, HGF, and VEGF, but not FGF2.

### HIF-1β-silencing suppresses tumor cell invasiveness and motility

**Figure 4:**
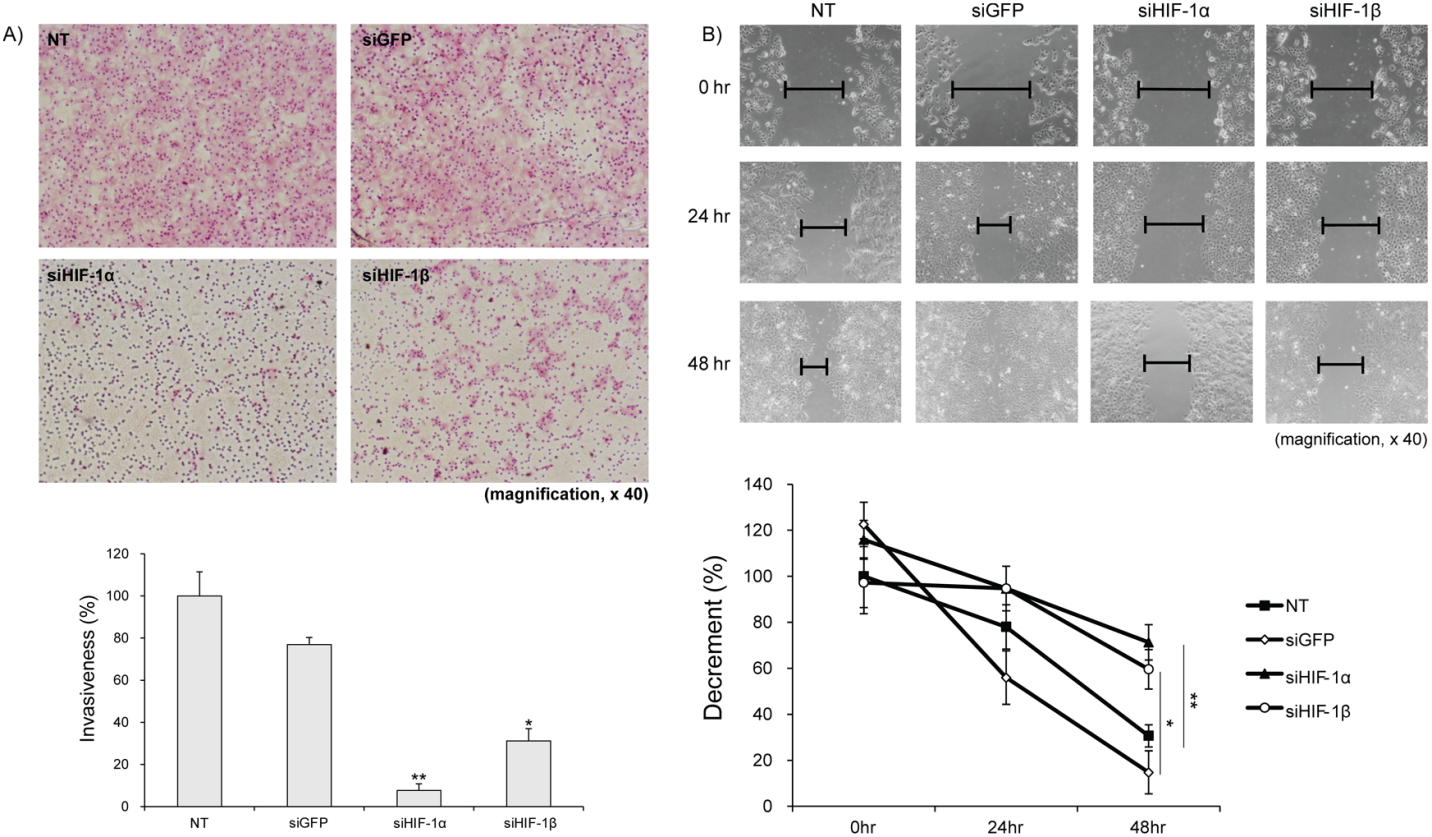
HIF-1β-silencing suppresses tumor cell invasiveness and migration ability. (A) Compared to control, invasiveness of tumor cell was significantly reduced in Hep3B cells transfected with siHIF-1α or **-**1β as demonstrated by transwell assays. (B) As compared to control, markedly reduced migration of HIF-1α- or **-**1β-silenced Huh-7 cells was confirmed by scratch and wound healing assay. NT, non-target; siGFP, siRNA against green fluorescent protein; siHIF1-α, siRNA against HIF-1α; siHIF-1β, siRNA against HIF-1β.

Since tumor cells exhibit potential to mobilize and invade into adjacent and distant regions, the effect of HIF-1β-silencing on invasiveness and migration ability of tumor cells was studied using various HCC cell lines. The effect of HIF-1β-silencing on the invasiveness of tumor cells was evaluated by assessing the number of cells that have mobilized and moved across the matrigel-coated trans-well to the gelatin coated-bottom well. Compared to control, siHIF-1β-silenced cells showed remarkably reduced number of cells in the bottom well, denoting suppression of tumor cell invasiveness (Fig. 4A). Likewise, as compared to control, markedly diminished migration of HIF-1β-silenced Huh-7 cells was confirmed by scratch and wound healing assay (Fig. 4B). Of note, silencing of HIF-1α also induced suppression of tumor cell invasiveness and migration. Together, these findings suggest that silencing of HIF-1β affects the invasiveness and migration of tumor cells under hypoxic conditions.

### Silencing of HIF-1β sensitizes tumor cells to hypoxic apoptosis

**Figure 5:**
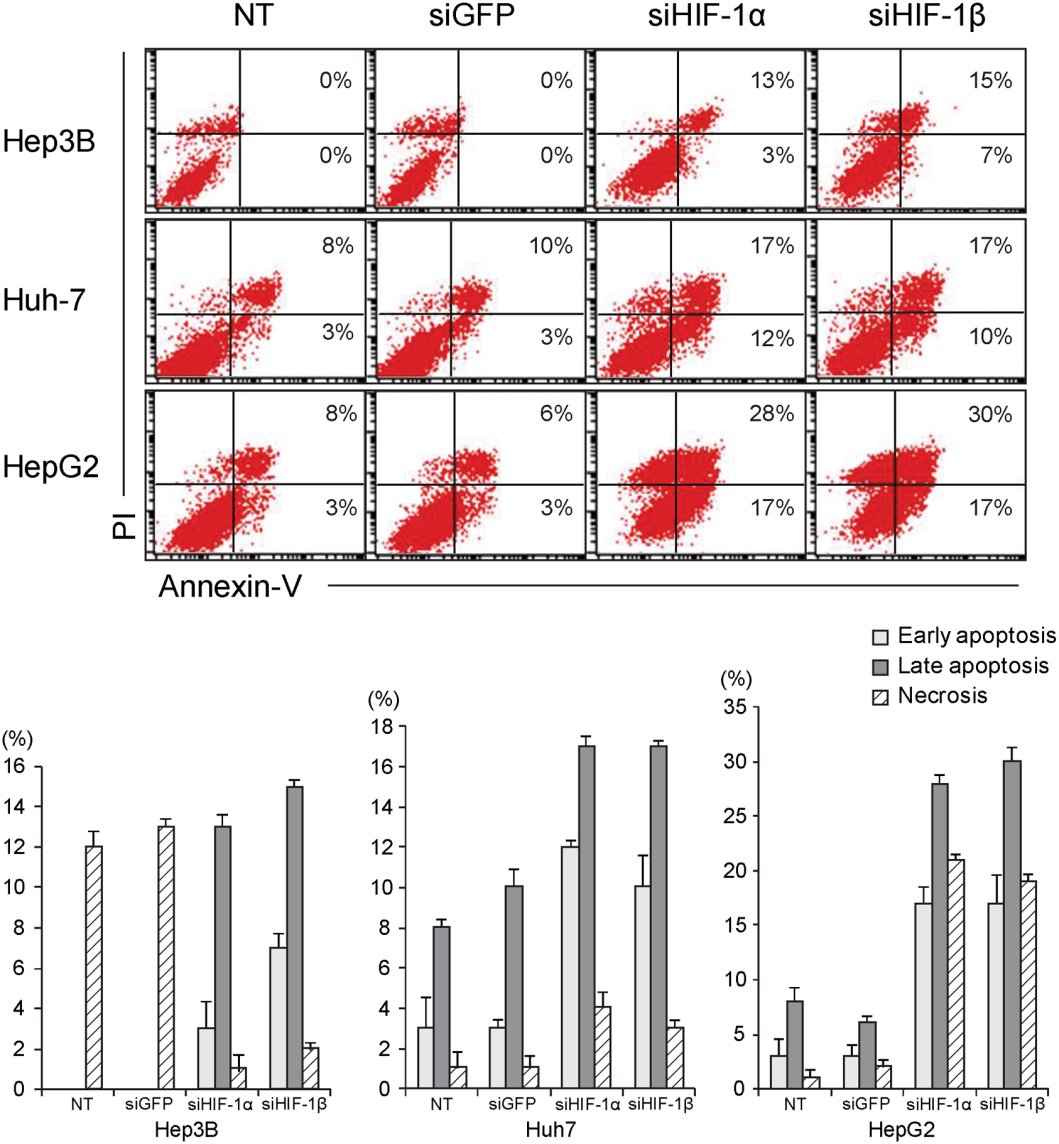
Silencing of HIF-1β sensitizes tumor cells to hypoxic apoptosis. Cell death assay demonstrated that hypoxic apoptosis was markedly increased by silencing HIF-1β in tumor cells, a finding similar to the effect of HIF-1α-silencing on tumor cell survival. NT, non-target; siGFP, siRNA against green fluorescent protein; siHIF1-α, siRNA against HIF-1α; siHIF-1β, siRNA against HIF-1β; PI, propidium iodide.

Normally, cells are predisposed to apoptosis upon exposure to prolonged hypoxic environment, a phenomenon known as hypoxic apoptosis. However, tumor cells tend to circumvent apoptosis through various mechanisms, with the aid of the HIF system. To evaluate whether HIF-1β is responsible for the resistance to hypoxic apoptosis, cell death assay was performed in HCC cells silenced for HIF-1α or HIF-1β followed by exposure to hypoxic environment. Cell death assay demonstrated that hypoxic apoptosis was merely induced in the control (Figure 5). On the contrary, hypoxic apoptosis was markedly increased by silencing HIF-1β, a finding similar to the effect of HIF-1α-silencing on tumor cell survival. Collectively, these data suggest that HIF-1β, along with HIF-1α, regulates hypoxic apoptosis of tumor cells under hypoxic conditions.

## Discussion

In the tumors, hypoxia is an important mechanism that induces proliferation, metastasis, and neovascularization of tumors [9], [13], [14]. HIF-1α is a key molecule in hypoxia [12], and is known to be involved in the proliferation of tumors and survival mechanisms such as angiogenesis and anti-apoptosis [15]. HIF-1α is highly regulated by oxygen concentration [12]. Under hypoxia, HIF-1α maintained and concentrated in the cytoplasm [10]. In normoxia, the HIF-1α proteins are rapidly degraded, resulting in essentially no detectable HIF-1α protein [15]. On the other hand, HIF-1β is not affected by oxygen concentration [18]. Although HIF-1α can function by forming a dimer with HIF-1β, few studies of HIF-1β have been performed [15]–[18]. HIF-1β was initially known as an ARNT factor, and plays an important role in the differentiation and development of many cell types such as T cells, neurons, and hepatocytes, by dimerization with AhR, which is similar to HIF-1α [18]–[20]. Moreover, the AhR pathway, which involves HIF-1β (ARNT), is known to mediate anti-tumor effects [21]. Among high-risk patients with HCC in one study, HIF-1β expression was high and was associated with cell cycle arrest [22]. We investigated the function of HIF-1β under hypoxic conditions. The hypoxia response, which plays a role in tumor development, presumably prioritizes the function of HIF-1β rather than that of ARNT. Accordingly, further study of HIF-1β is warranted.

This study proceeded under the assumption that even when dimerization of HIF-1α and HIF-1β is inhibited, compared with inhibition of HIF-1α expression, the same result as expression of HIF-1α can be obtained. As a result, siRNA inhibition of HIF-1α and siRNA inhibition of HIF-1β were treated at equal densities, and the present study performed the MTT assay to determine cell growth when a hypoxic stimulus was applied. Effect of inhibition for tumor growth similar to case of inhibition for HIF-1α could be identified.

Based on the effect of tumor inhibition obtained from the MTT assay, expression of the growth factors EGF, HGF, and VEGF related to tumor growth through real-time quantitative PCR, Western blotting, and ELISA was decreased [4], [14]. EGF is a growth factor that stimulates cell growth, proliferation, and differentiation by binding to its receptor EGFR. HGF regulates cell growth, cell motility, and morphogenesis by activating a tyrosine kinase signaling cascade after binding to the proto-oncogenic c-Met receptor. VEGF is a signaling protein produced by cells that stimulates neo-vasculogenesis and angiogenesis [23]. The above three factors are all related to tumor growth and development, and control of the expression of these three growth factors plays an important role in anti-tumor effects. Based on these findings, the expression of HIF-1β is inhibited and this subsequently inhibits the formation of a dimer with HIF-1α. Therefore, expression control of a sub-gene is another method to inhibit the activation of tumors under hypoxic conditions.

In the present study, the influence of hypoxia on cells and that of HIF-1β knockdown were evaluated using wound healing assay and tumor cell invasion. When HIF-1β expression was inhibited, wound healing was suppressed; similarly, when HIF-1α was knocked down, motility decreased. Moreover, among various liver cancer cell lines, only the Huh7 cell line can be used to assess tumor invasion by measuring the degree of tumor invasiveness that was performed using Huh7 in vitro. Further investigation is needed to determine the cause of such a difference. The current study evaluated the invasion degree of Huh7 cells when expression of HIF-1β was inhibited, and the result was compared with the group for which the expression of HIF-1α was inhibited. When the expression of HIF-1α was inhibited, invasion did not occur as expected. We guessed that inhibition of HIF-1β expression prevented the formation of a dimer with HIF-1α; in turn, various functions that should be performed under hypoxic conditions, such as inhibited cell survival, cell motility, cell growth, and impeded invasion.

Regarding tumor proliferation, a hypoxic state is important for tumor growth to start. It is thought that HIF-1 expression (HIF-1α and HIF-1β) controls the initiation of tumor growth, and can be important in affecting anti-tumor growth by changing growth to be more malignant in a hypoxic state. Further study is required to determine other possible functions of HIF-1β that are comparatively less known than those of HIF-1α, which has drawn most of the attention until now.

## Supporting Information

File S1 **Suppression of tumor cell growth by knockdown of hypoxia-inducible factors-1α.** Tumor cell growth after knockdown of HIF-1α was measured by MTT assay. Tumor cells were susceptible to growth inhibition under hypoxic conditions when more than 100 nM of siHIF-1α was transfected. However, normoxic conditions (100 nM) did not show significant difference.
(TIF)

File S2 **Confirmation of dimerization with HIF-1α and HIF-1β by Immunoprecipitation.** HIF-1α was bounded with mouse anti-HIF-1α using IgG beads. siHIF-1α or siHIF-1β group weakly detect HIF-1α or HIF-1β band. But, HIF-1α/HIF-1β strongly expressed over 2∼3 times in the control group.
(TIF)

## References

[1] Greer SN, Metcalf JL, Wang Y, Ohh M (2012) The updated biology of hypoxia-inducible factor. The EMBO journal 31: 2448–2460. 22562152 10.1038/emboj.2012.125 PMC3365421

[2] Ke Q, Costa M (2006) Hypoxia-inducible factor-1 (HIF-1). Molecular pharmacology 70: 1469–1480. 16887934 10.1124/mol.106.027029

[3] Semenza GL (2007) Hypoxia-inducible factor 1 (HIF-1) pathway. Science’s STKE: signal transduction knowledge environment 2007: cm8. 10.1126/stke.4072007cm8 17925579

[4] Zhong H, Chiles K, Feldser D, Laughner E, Hanrahan C, et al (2000) Modulation of hypoxia-inducible factor 1alpha expression by the epidermal growth factor/phosphatidylinositol 3-kinase/PTEN/AKT/FRAP pathway in human prostate cancer cells: implications for tumor angiogenesis and therapeutics. Cancer research 60: 1541–1545. 10749120

[5] Hankinson O (1995) The aryl hydrocarbon receptor complex. Annual review of pharmacology and toxicology 35: 307–340. 10.1146/annurev.pa.35.040195.001515 7598497

[6] Reisz-Porszasz S, Probst MR, Fukunaga BN, Hankinson O (1994) Identification of functional domains of the aryl hydrocarbon receptor nuclear translocator protein (ARNT). Molecular and cellular biology 14: 6075–6086. 8065341 10.1128/mcb.14.9.6075-6086.1994 PMC359134

[7] Dougherty EJ, Pollenz RS (2008) Analysis of Ah receptor-ARNT and Ah receptor-ARNT2 complexes in vitro and in cell culture. Toxicological sciences: an official journal of the Society of Toxicology 103: 191–206. 18096572 10.1093/toxsci/kfm300 PMC2396590

[8] Nukaya M, Walisser JA, Moran SM, Kennedy GD, Bradfield CA (2010) Aryl hydrocarbon receptor nuclear translocator in hepatocytes is required for aryl hydrocarbon receptor-mediated adaptive and toxic responses in liver. Toxicological sciences: an official journal of the Society of Toxicology 118: 554–563. 20935161 10.1093/toxsci/kfq305 PMC2984536

[9] Pillai R, Huypens P, Huang M, Schaefer S, Sheinin T, et al (2011) Aryl hydrocarbon receptor nuclear translocator/hypoxia-inducible factor-1{beta} plays a critical role in maintaining glucose-stimulated anaplerosis and insulin release from pancreatic {beta}-cells. The Journal of biological chemistry 286: 1014–1024. 21059654 10.1074/jbc.M110.149062 PMC3020708

[10] Wang GL, Jiang BH, Rue EA, Semenza GL (1995) Hypoxia-inducible factor 1 is a basic-helix-loop-helix-PAS heterodimer regulated by cellular O_2_ tension. Proceedings of the National Academy of Sciences of the United States of America 92: 5510–5514. 7539918 10.1073/pnas.92.12.5510 PMC41725

[11] Yun CO, Kim E, Koo T, Kim H, Lee YS, et al (2005) ADP-overexpressing adenovirus elicits enhanced cytopathic effect by induction of apoptosis. Cancer gene therapy 12: 61–71. 15375379 10.1038/sj.cgt.7700769

[12] Maxwell PH, Dachs GU, Gleadle JM, Nicholls LG, Harris AL, et al (1997) Hypoxia-inducible factor-1 modulates gene expression in solid tumors and influences both angiogenesis and tumor growth. Proceedings of the National Academy of Sciences of the United States of America 94: 8104–8109. 9223322 10.1073/pnas.94.15.8104 PMC21564

[13] Roessler S, Budhu A, Wang XW (2007) Future of molecular profiling of human hepatocellular carcinoma. Future oncology 3: 429–439. 17661718 10.2217/14796694.3.4.429

[14] Wang L, Park H, Chhim S, Ding Y, Jiang W, et al (2012) A novel monoclonal antibody to fibroblast growth factor 2 effectively inhibits growth of hepatocellular carcinoma xenografts. Molecular cancer therapeutics 11: 864–872. 22351746 10.1158/1535-7163.MCT-11-0813 PMC3324641

[15] Pugh CW, Ratcliffe PJ (2003) Regulation of angiogenesis by hypoxia: role of the HIF system. Nature medicine 9: 677–684. 10.1038/nm0603-677 12778166

[16] Chilov D, Camenisch G, Kvietikova I, Ziegler U, Gassmann M, et al (1999) Induction and nuclear translocation of hypoxia-inducible factor-1 (HIF-1): heterodimerization with ARNT is not necessary for nuclear accumulation of HIF-1alpha. Journal of cell science 112 (Pt 8): 1203–1212. 10.1242/jcs.112.8.1203 10085255

[17] Pollenz RS, Sattler CA, Poland A (1994) The aryl hydrocarbon receptor and aryl hydrocarbon receptor nuclear translocator protein show distinct subcellular localizations in Hepa 1c1c7 cells by immunofluorescence microscopy. Molecular pharmacology 45: 428–438. 8145729

[18] Sogawa K, Nakano R, Kobayashi A, Kikuchi Y, Ohe N, et al (1995) Possible function of Ah receptor nuclear translocator (Arnt) homodimer in transcriptional regulation. Proceedings of the National Academy of Sciences of the United States of America 92: 1936–1940. 7892203 10.1073/pnas.92.6.1936 PMC42397

[19] Wood SM, Gleadle JM, Pugh CW, Hankinson O, Ratcliffe PJ (1996) The role of the aryl hydrocarbon receptor nuclear translocator (ARNT) in hypoxic induction of gene expression. Studies in ARNT-deficient cells. The Journal of biological chemistry 271: 15117–15123. 8662957 10.1074/jbc.271.25.15117

[20] Wang Y, Li Y, Wang D, Li Y, Chang A, et al (2012) Suppression of the hypoxia inducible factor-1 function by redistributing the aryl hydrocarbon receptor nuclear translocator from nucleus to cytoplasm. Cancer letters 320: 111–121. 22306343 10.1016/j.canlet.2012.01.037 PMC3319868

[21] Shi S, Yoon DY, Hodge-Bell K, Huerta-Yepez S, Hankinson O (2010) Aryl hydrocarbon nuclear translocator (hypoxia inducible factor 1beta) activity is required more during early than late tumor growth. Molecular carcinogenesis 49: 157–165. 19824022 10.1002/mc.20585 PMC2938742

[22] Liang Y, Li WW, Yang BW, Tao ZH, Sun HC, et al (2012) Aryl hydrocarbon receptor nuclear translocator is associated with tumor growth and progression of hepatocellular carcinoma. International journal of cancer Journal international du cancer 130: 1745–1754. 21544813 10.1002/ijc.26166

[23] Yamaguchi R, Yano H, Iemura A, Ogasawara S, Haramaki M, et al (1998) Expression of vascular endothelial growth factor in human hepatocellular carcinoma. Hepatology 28: 68–77. 9657098 10.1002/hep.510280111

