# Supplementary figures and images for: Silencing of Hypoxia-Inducible Factor-1β Induces Anti-Tumor Effects in Hepatoma Cell Lines under Tumor Hypoxia
Source: PLoS One. 2014 Jul 28;9(7):e103304. doi: 10.1371/journal.pone.0103304 (PMC4113399; doi:10.1371/journal.pone.0103304)

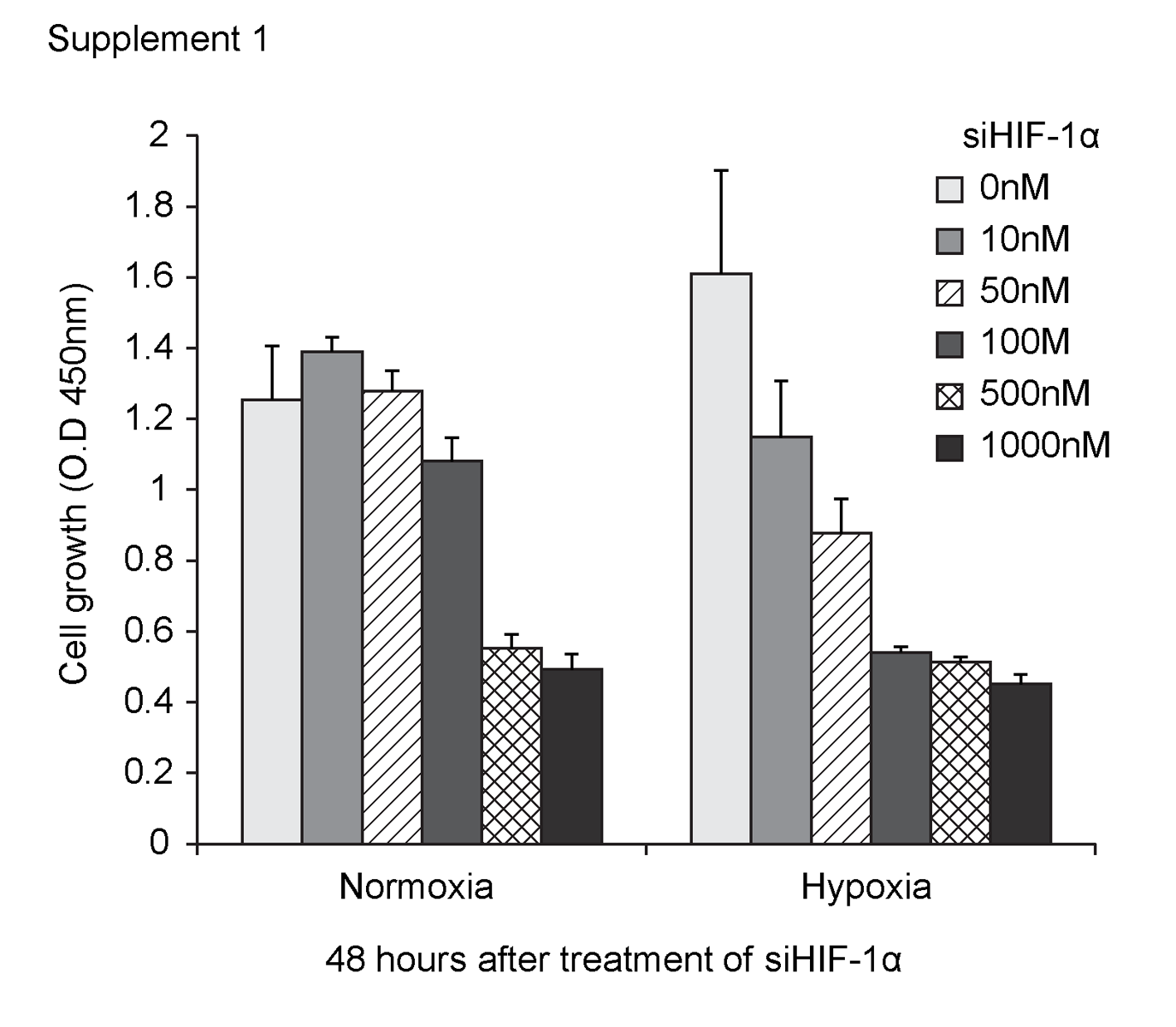

Supplement: File S1 — Suppression of tumor cell growth by knockdown of hypoxia-inducible factors-1α. Tumor cell growth after knockdown of HIF-1α was measured by MTT assay. Tumor cells were susceptible to growth inhibition under hypoxic conditions when more than 100 nM of siHIF-1α was transfected. However, normoxic conditions (100 nM) did not show significant difference. (TIF) [file pone.0103304.s001.tif]

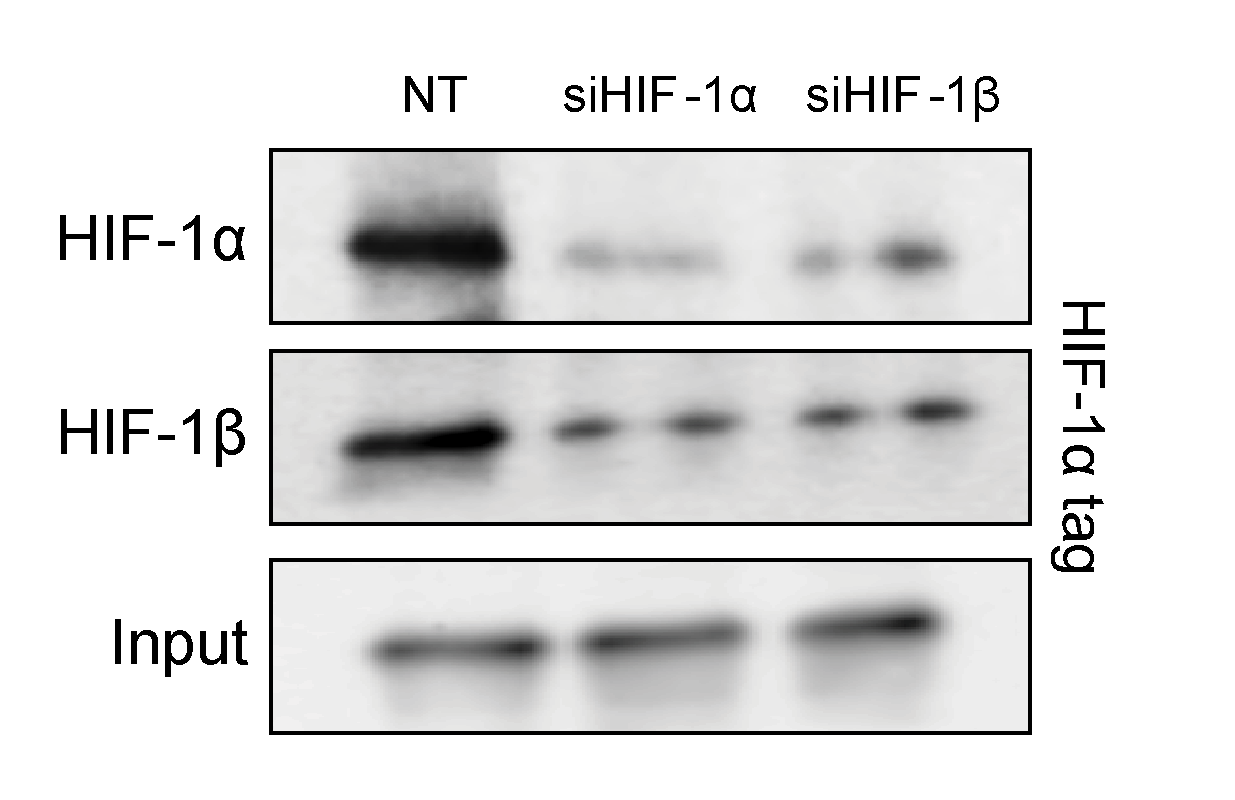

Supplement: File S2 — Confirmation of dimerization with HIF-1α and HIF-1β by Immunoprecipitation. HIF-1α was bounded with mouse anti-HIF-1α using IgG beads. siHIF-1α or siHIF-1β group weakly detect HIF-1α or HIF-1β band. But, HIF-1α/HIF-1β strongly expressed over 2∼3 times in the control group. (TIF) [file pone.0103304.s002.tif]
